# Molecular and biological functions of resveratrol in psychiatric disorders: a review of recent evidence

**DOI:** 10.1186/s13578-020-00491-3

**Published:** 2020-11-07

**Authors:** Mehran Shayganfard

**Affiliations:** Department of Psychiatry, Arak University of Medical Sciences, Arak, Iran

**Keywords:** Resveratrol, Anxiety, Depression, Schizophrenia

## Abstract

Mental disorders including depression, anxiety, schizophrenia, autism spectrum disorders, bipolar and etc. have a considerable proportion of global disorder burden. Many nutritional psychiatry investigations have been conducted to evaluate the relationship between several individual nutrients such as herbal compounds with mental health. Resveratrol, a famous polyphenol compound, is known as an antioxidant, anti-inflammatory, anti-apoptotic, and neuroprotective agent regulating the function of brain and improves the behavioral factors associated with learning, anxiety, depression, and memory. In addition, this natural compound can cross the blood–brain barrier representing neurological influences. The pharmacological interest of utilizing resveratrol in mental disorders is due to its anti-inflammatory and antioxidant features. The aim of this paper was to review the studies evaluated the potential effects of resveratrol on mental disorders.

## Introduction

Mental disorders including depression, anxiety, schizophrenia, autism spectrum disorders (ASD), bipolar and etc. have a considerable proportion of global disorder burden [1]. Alterations and shifting of life styles from traditional features by globalization and urbanization are related to an elevation in mental disorders [2]. In recent decades, diet has been indicated to play an important role in both physical and mental disorders incidence and treatment [3]. Several associated processes showing the relationship between diet and mental disorders have been found in emerging evidence [4]. Many nutritional psychiatry investigations have been conducted to evaluate the relationship between several individual nutrients such as vitamins, minerals, omega-3, and herbal compounds with mental health [5].

Resveratrol (3,4′,5-trihydroxystilbene; C14H12O3; MW 228.247 g/mol) is a polyphenolic compound existing in berries, grapes, peanuts, and wine. Resveratrol is known as an antioxidant, anti-inflammatory, anti-apoptotic, and anti-tumor agent [6–8]. Moreover, this natural compound has been reported as a neuroprotective agent. Additionally, evidence has been indicated that resveratrol is able protect both neuronal and glial cells from damages [9]. Interestingly, resveratrol regulates the function of brain and improves the behavioral factors associated with learning, anxiety, depression, and memory [10, 11]. Resveratrol has a fast metabolism forming glucuronide and sulfate derivatives excreted through urine. Resveratrol can be detected in the blood at the concentrations range of nanomolar [12]. Recent research reported that the highest blood and brain levels of resveratrol can be detected about 20–30 min after oral administration maintaining up to 60 min [13]. Thus, resveratrol can cross the blood–brain barrier representing neurological influences. The pharmacological interest of utilizing resveratrol in mental disorders is due to its anti-inflammatory feature in addition of its potential effects on these diseases [14]. Therefore, the combined effects represented by resveratrol have taken the attention of researchers investigating the novel drugs for treatment of mental disorders. The aim of this paper was to review the studies evaluated the potential effects of resveratrol on mental disorders.

## Neuroprotective features of resveratrol

Resveratrol has low bioavailability due to an extensive intestinal and hepatic metabolism of its oral administration [15]. Considerably, numerous studies have indicated that resveratrol can cross the blood brain barrier. For instance, a recent study reported the levels of resveratrol in the brain, 4 h after intraperitoneal injection [16]. In another investigation, chronic intake of resveratrol was found to protect hippocampus of rodents from kainic acid [17]. The effect of oral administration of resveratrol on cognitive performance in either healthy people or patients with cognitive disorders have been investigated in limited literature. An investigation assessed vasodilatory function of resveratrol on blood flow improvement to promote cognitive action. The authors found that oral intake of resveratrol led to dose-dependent elevations in cerebral blood flow during cognitive function activating the frontal cortex [18]. Another study showed that resveratrol was able to increase endothelium dependent vasodilation and flow-mediated dilation of brachial artery enhancing cerebrovascular and cognitive performance [19].

Excessive amounts of reactive oxygen species (ROS) were reported to be related to cognitive deficits [20]. The antioxidant function of resveratrol has been proved in numerous studies due to its scavenging activity of free radicals as well as upregulating effects on antioxidant enzymes such as glutathione peroxidase [21, 22]. In addition, resveratrol is able to protect hippocampal neurons exposed to nitric oxide [23]. Interestingly, resveratrol play role in the cellular response by regulating the enzymes acting in stress response such as quinone reductase2 [24]. Quinone reductase2 has been shown to be upregulated in the hippocampus involved in learning and memory proposing that the elevated levels of this enzyme may enhance memory impairments [25]. Recent evidence showed that resveratrol suppressed quinone reductase2 leading to elevation of cellular resistance against neural death mediated by oxidative stress [26]. Moreover, resveratrol is able to induce heme oxygenase 1 enhancing resistance against oxidative stress and neural damage [27]. Furthermore, resveratrol activates AMP-activated protein kinase (AMPK) regulating cell survival in oxidative stress response [28]. Additionally, resveratrol is able to protect mitochondria from oxidative stress [29]. Besides, microglial activation enhances neural death followed by brain damage by producing neurotoxic pro-inflammatory factors [30]. Resveratrol has been detected to reduce the pro-inflammatory factors such as cyclooxigenase1 (COX1) involved in cytokines production [22]. Pro-inflammatory production can be decreased by resveratrol via inhibiting nuclear factor kappaB (NF-kB) and activator protein- (AP-1) [31]. Moreover, resveratrol can reduce prostaglandins, NO, and tumor necrosis factor-α (TNF-α) [32–34].

## The pathogenesis of mental disorders

### Depression

Major depression is known as one of the public health problems and a common psychiatric disease. It has been estimated to have 121 affected people entire the world [35]. Additionally, severe depression leads to a high morbidity and economic as well as social consequences [36]. Cognitive impairments as well as neurocognitive deficits of processing speed, learning, memory, executive performance, and attention are presented in patients suffering from depression [37, 38]. The exact mechanisms of pathogenesis of this disorder are not clear yet, however some factors are shown to be associated with depression progression including genetic factors and non-genetic factors [39]. Non-genetic factors such as stress, emotional trauma, viral infection and neuro-related abnormalities may be complicated in the pathogenesis of depression [36]. An impairment in monoamine neurotransmitter system such as decreasing of serotonin, dopamine, and noradrenaline as well as neural damage and neural cell loss also have been reported to take role in the pathogenesis of depression [40, 41]. Several studies proposed that mitochondrial dysfunction leads to cellular damage in depression [42, 43]. Besides, it has been indicated that mitochondrial alterations may produce less ATP, reduced activities of enzymes involved in respiratory chain, and decreased physiological respiratory rate as well as capacity of electron transport chain in patients with depression [44, 45].

A large amounts of studies found the role of oxidative stress in the pathogenesis of depression [46]. In a recent investigation, increased concentrations of malondialdehyde (MDA), and therefore lipid peroxidation were reported in depressive patients [47, 48]. Similarly, another study found increased lipid peroxidation represented by elevated levels of F(2)-isoprostanes in patients suffering from depression [49, 50]. 8-Hydroxydeoxyguanosine (8-OHdG) which is an indicator for oxidative stress to DNA, was elevated in depressive people [51]. In addition, various clinical trials and in vivo studies observed that SOD activity elevated in depression [52]. Furthermore, antioxidant molecules such as vitamin E and vitamin C were shown to be decreased in depressive individuals in comparison to healthy people [53, 54]. Besides, the severity of depression disorder has a positive relationship with the levels of oxidative stress markers [55]. Elevated blood concentrations and gene expressions of pro-inflammatory cytokines including interleukin (IL)-1, IL-6, IL-8, interferon-γ (IFN-γ) and tumor necrosis factor-α (TNF-α) were demonstrated to be associated with depression [56, 57].

### ASD

ASD are heterogeneous diseases characterized by restrictive behavior as well as communication and social interaction impairments [58]. Currently, the exact pathogenesis of ASD is not fully understood. Several abnormalities related and non-related to the central nervous system (CNS) have been proposed to be complicated in ASD pathogenesis leading to various cognitive and behavioral impairments [59–61]. ASD has been assumed to result from a group of systemic dysfunctions more than a specific organ abnormality [62]. In recent decades, researchers attempted to find genetic factors such as chromosomal abnormalities or a single gene related to ASD incidence but no exact relationship has been found yet [63]. Besides, recent investigations have been aimed to find gene-environment interaction, epigenetic factors, and pathophysiological process to explain the etiology and development of ASD. Currently, multiple factors including inflammation, oxidative stress, immune dysfunction, mitochondrial dysfunction, and environmental toxicities have been reported to be involved in psychiatric disorders like ASD [64, 65]. Neuro-inflammation characterized by CNS-specific and chronic glial reactions has been identified to take role in ASD pathogenesis. Neuro-inflammation leads to neuron growth abnormality, increased production of pro-inflammatory cytokines, and plaque generation and therefor damages brain tissue [66]. Environmental toxicants such as pesticides, heavy metals, and chemicals can affect biochemical mechanisms leading to increased oxidative stress, decreased glutathione and dysregulated cellular signaling pathways all of which can impair cell functions [67]. Another factor known to be involved in ASD pathogenesis is oxidative stress characterized by ROS-related cell damage [61]. Moreover, mitochondrial dysfunction is also complicated in many psychiatric disorders such as schizophrenia, bipolar, depression, dementia, and ASD [68, 69]. Mitochondrial dysfunction affect not only cell energy production but also other mitochondria-dependent processes such as programmed cell death or apoptosis, synaptic plasticity, calcium homeostasis, and neurotransmitter release [70, 71].

### Schizophrenia

About 1 per cent of people suffer from schizophrenia, a chronic psychiatric disease, entire the world. This disorder initiates in early adulthood or late adolescence with psychotic symptoms containing positive, negative and cognitive symptoms [72]. Positive symptoms are those which are not represent in healthy people but are developed in individuals suffering from schizophrenia. These symptoms include paranoia, delusions, major thought disorder, visual and auditory hallucinations. Negative symptoms present in normal people but are decreased or disappeared in patients with schizophrenia including apathy, alogia, social withdrawal, anhedonia, and behavioral perseveration. Memory impairment, unstable attention, and executive activities disturbances are known as cognitive symptoms of schizophrenia. Recently, various neuro-immune factors have been detected to be complicated in the etiology and onset of neuropsychiatric diseases especially those developed during early brain formation such as schizophrenia [73]. Thus, immunological mechanisms targeting brain development are proposed to affect the brain and behavioral functions [74, 75]. Cytokines and their receptors have been observed to be highly expressed by neural and glial cells during the development of fetal brain suggesting to be effective on neurodevelopmental processes and neural survival [76]. Therefore, their abnormal amounts early brain development may affect neurodevelopmental mechanisms negatively leading to increased risk of the incidence of disorders with developmental origins like schizophrenia. Many investigations showed that anti-inflammatory and immunosuppressive interventions could decreased inflammation in schizophrenia [77, 78]. For instance, IL-10 production was seen to be higher in schizophrenia patients [79]. In addition, IL-1 gene complex has been suggested to play role in schizophrenia progression [80]. Thus, immune dysfunction along with chronic inflammation have been suggested to be relevant with infection-associated pathogenic processes in schizophrenia. Inflammatory response against postnatal environmental factors such as stress or drug abuse may impair brain maturation enhancing the incidence and development of chronic schizophrenia [81, 82]. Thus, it has been suggested that anti-inflammatory drugs may be able to delay the onset and progression of schizophrenia especially in the primary phases of the disease [83].

### The therapeutic effects of resveratrol on mental disorders

resveratrol has attracted researchers’ attention as a potential compound for treatment of several mental diseases. We summarized the therapeutic functions of resveratrol in several pathways complicated in mental disorders including depression, anxiety, ASD, and schizophrenia as mental disorders.

### Depression

#### The effects of resveratrol on oxidative stress, inflammation, and apoptosis

Chronic stress is a routine feature of our life inducing depression-like behaviors via alterations of proteins and some pathways in the brain. Resveratrol was found to represent anti-depressant effects. Numerous evidence indicated the potential roles of oxidative stress, inflammation, and apoptosis in the pathogenesis of depression. In an in vivo study, depression like behaviors induced in rats by submitting to 8 weeks of chronic unpredictable mild stress (CUMS) leading to decreased expressions of β-catenin, Bcl-2, GSH, and total antioxidant (TAC) as well as increased expressions of glycogen synthase kinase-3 β (GSK-3β), nuclear factor-kappa B (NF-kB), TNF-α, IL-1β, and MDA levels in hippocampus. Considerably, resveratrol administration improved all of these effects by inhibiting the neuro-inflammation, apoptosis, and oxidative stress as well as increasing hippocampal brain-derived neurotrophic factor (BDNF) and β-catenin concentrations [84]. Another work examined the role of resveratrol in ameliorating of ovariectomy-induced anxiety and depression-like behaviors in female mice. Resveratrol was able to reduce the depression and anxiety of the mice by attenuating activation of microglia and the NF-kB and NLRP3 in the hippocampus by increasing Sirt1 levels. Thus, the authors concluded that resveratrol is able to ameliorate the psycho-behavioral alterations induced by estrogen deficiency through suppression of inflammatory mechanisms and to be effective in the alleviation of postmenopausal changes [85]. The effects of resveratrol on testicular dysfunction associated with CUMS-induced depressive-like behaviors was assessed in an in vivo study. CUMS-induced depression led to elevated serum levels of corticosterone, decreased serum and hippocampus levels of serotonin, and reduced hippocampal levels of BDNF. In addition, CUMS reduced testosterone concentrations, increased testicular expressions of NF-kB, TNF-α, IL-1β and Bax as well as increased the expression of Bcl-2. CUMS also increased the oxidative stress in testis. Resveratrol administration presented testicular protective effects by inhibiting oxidative stress, apoptosis and inflammation resulting in elevation of serum levels of testosterone and β-catenin [86]. Wang et al., [7] investigated the anti-depressant effects of resveratrol in rat models with chronic restraint stress (CRS)—induced depression-like behaviors. The findings indicated that resveratrol administration remarkably attenuated the CRS-induced depressant behaviors. Resveratrol increased BDNF levels, and Bcl-2 expression in hippocampus. Additionally, resveratrol decreased Bax mRNA expression.

Increasing evidence showed that energy deficiency could be involved in the development of depression. Mitochondrial damage is the main cause of energy deficiency which may result in abnormalities in synaptic neurotransmission and therefor depression. Resveratrol was detected to increase hippocampal ATP levels, mitochondrial DNA, the expressions of SIRT1 and peroxisome proliferator-activated receptor γ coactivator (PGC)1α as well as decrease Na^+^-K^+^-ATPase and pyruvate levels. Moreover, resveratrol elevated serotonin levels and decreased its transporter, SERT, in the hippocampus [87]. Chen et al., [88] examined the influence of resveratrol on lipopolysaccharide (LPS)-induced depression-like behavior mice. They showed that resveratrol improved LPS-induced depression as well as apoptosis mitochondrial oxidative stress in the hippocampus. The effect of resveratrol on neurogenesis related to depression was determined in an investigation. Resveratrol treatment enhanced neurogenesis, upregulated Sirt1, and inhibited NF-kB activation ameliorating depression-like behaviors [89]. Moreover, resveratrol was reported to be able to increase neurotransmitters dopamine and serotonin significantly in the prefrontal cortex and NPY expression in the brain suggesting to act as an antagonist for depression treatment [90].

#### The effect of resveratrol on Wnt/βcatenin pathway

The phosphoinositide 3 kinase and protein kinase B (PI3K/Akt) signaling pathway is a process regulating critical cellular pathways including inflammation, growth and survival. Akt phosphorylation leads to the phosphorylation of glycogen synthase kinase-3β(GSK3β) inhibiting GSK3β activity and effecting on its substrates including Bax, NF-κB, TNF-α, IL-6, tau protein, and c-Jun transcript [91, 92]. Recent evidence has indicated that the activity of GSK3β maybe elevated in animals with chronic stress. Moreover, the suppression of GSK3β activity decreased chronic stress induced neuronal apoptosis [93]. In addition, GSK3β polymorphisms has been shown to be related to major depressive diseases [94]. Chronic stress also leads to an imbalance between Bax and Bcl-2 protein expressions which are pro-apoptotic and anti-apoptotic parameters, respectively. GSK-3β regulates β-catenin phosphorylation and degradation inhibiting the canonical Wnt pathway activation [95]. Recent investigations reported that prevention of GSK-3β might ameliorate depression [96]. increased activity of GSK-3β was found in depressed suicide victims [97]. evidence suggests that resveratrol can ameliorate the decreased ratio of p-GSK-3β/GSK-3β [98].

According to evidence, subclinical hypothyroidism (SCH) has a close relationship with depression-like behavior. The aim of a recent work was to determine the antidepressant effect of resveratrol on rat model with SCH. Rats received intra-gastric injection of resveratrol. The results demonstrated that resveratrol administration decreased plasma levels of thyroid-stimulating hormone (TSH) as well as hypothalamic mRNA levels of thyrotropin-releasing hormone (TRH). In addition, resveratrol treatment led to reduction in plasma levels of corticosterone and hypothalamic corticotrophin-releasing hormone (CRH) expression. Resveratrol also upregulated the protein levels and phosphorylation of GSK3β as well as cyclin D1 and c-myc. Moreover, this compound decreased phosphorylation of β-catenin in hippocampus. Thus, resveratrol presented antidepressant function through decreasing the hyperactivity of hypothalamic–pituitary–adrenal (HPA) axis and regulating the Wnt/β-catenin pathway and hypothalamic–pituitary–thyroid (HPT) axis [99]. The potential effects of resveratrol on HPA axis were confirmed by another work in which resveratrol led to a reduction in plasma levels of corticosterone and CRH expression in hypothalamus. This work also found that resveratrol decreased plasma levels of IL-6, CRP, TNF-α, BDNF, βcatenin phosphorylation whereas increased the phosphorylation of GSK-3β in hippocampus [100]. The findings of another study also showed that resveratrol could ameliorate depression by activation of the Akt/GSK3β pathway and regulation of pro-inflammatory cytokines and apoptosis [101]. Oxidative stress and neuro-inflammation also attenuated by resveratrol via NETRIN1–mediated extracellular signal–regulated kinase/cAMP signaling pathway representing antidepressant effects [102]. These studies propose a role for GSK-3β in the antidepressant features of resveratrol.

These studies evaluated efficacy of resveratrol for treatment of depression were ummarized in Table 1.

**Table 1:** Animal studies that investigated the therapeutic effects of resveratrol on depression

Disease	Dose of resveratrol	Model	Findings	Publish year	Refs.
Chronic unpredictable mild stress-induced depression	80 mg/kg	In vivo	Inhibited neuro-inflammation, oxidative stress, and apoptosis, increased hippocampal BDNF and β-catenin levels	2018	[84]
Ovariectomy-induced depression	20 mg/kg	In vivo	Decreased activation of microglia, NF-kB, and NLRP3 in hippocampus, increased levels of Sirt1	2019	[85]
Chronic unpredictable mild stress-induced depression	80 mg/kg	In vivo	Inhibited oxidative stress, apoptosis and inflammation elevation of serum levels of testosterone and β-catenin	2019	[86]
Chronic restraint stress- induced depression	80 mg/kg	In vivo	Increased BDNF levels, and Bcl-2 expression in hippocampus, decreased Bax mRNA expression	2016	[7]
Subclinical hypothyroidism	15 mg/kg	In vivo	Decreased TSH, TRH, corticoesterone, and CRH hormones, increased GSK3β phosphorylation, cyclin D1 and c-myc, decreased β-catenin phosphorylation, decreased activity of HPA axis	2016	[99]
Chronic unpredictable mild stress-induced depression	15 mg/kg/day	In vivo	Decreased corticosterone and CRH, increased IL-6, CRP, TNF-α, BDNF, βcatenin phosphorylation, decreased the phosphorylation of GSK-3β in hippocampus	2017	[100]
Chronic unpredictable mild stress-induced depression	40 or 80 mg/kg/day	In vivo	activated Akt/GSK3β pathway and regulated pro-inflammatory cytokines and apoptosis	2019	[101]
Ouabain-induced depression	10 mg/kg	In vitro/in vivo	Decreased oxidative stress and neuro-inflammation	2018	[102]
Chronic unpredictable mild stress-induced depression	50 mg/kg	In vivo	Increased hippocampal ATP levels, mitochondrial DNA, the expressions of SIRT1 and PGC1α, decreased Na^+^-K^+^-ATPase and pyruvate levels, increased serotonin levels, decreased SERT in the hippocampus	2020	[87]
LPS-induced depression	0.3 mg/kg	In vivo	Improved apoptosis and oxidative stress in hippocampus and depressant-like behaviors	2017	[88]
LPS-induced depression	20 mg/kg	In vivo	Enhanced neurogenesis, upregulated Sirt1, and inhibited NF-kB activation	2016	[89]
Chronic unpredictable mild stress-induced depression	10, 20 and 30 mg/kg	In vivo	Increased dopamine and serotonin in the prefrontal cortex and increased NPY expression in the brain	2019	[90]

**Fig. 1:**
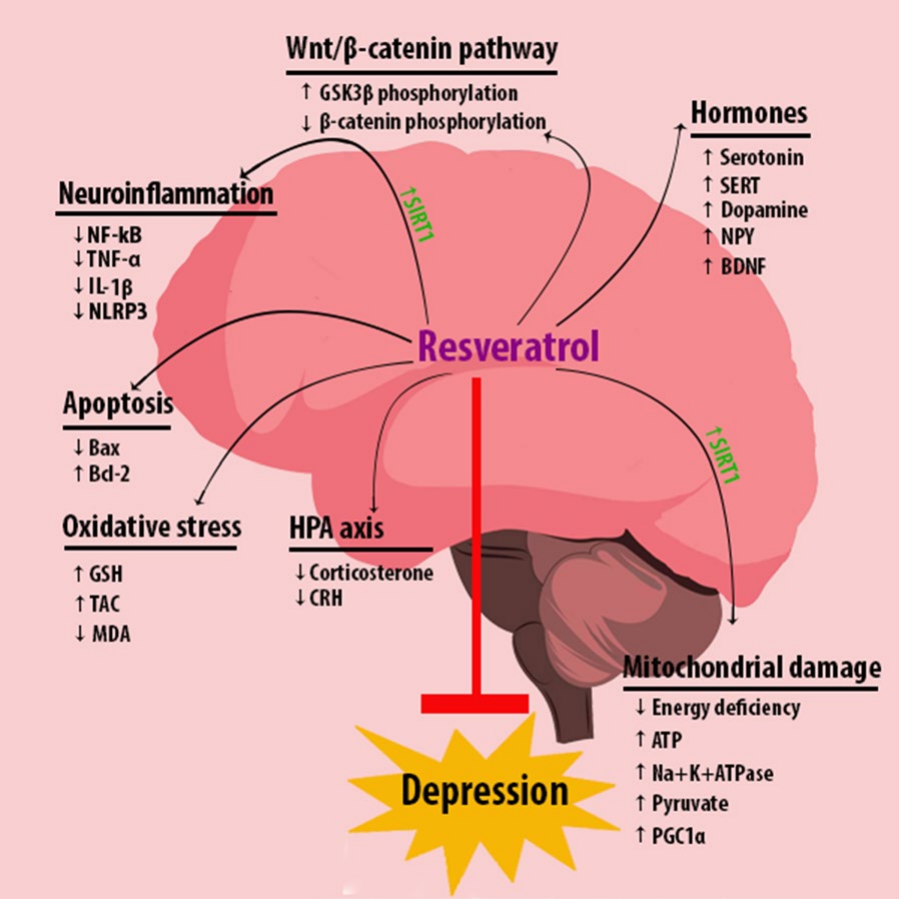
A schematic representation of resveratrol function in the treatment of Depression

The theraupetic targets of resveratrol in depression were shown in Fig. 1.

### Anxiety disorder

#### The effect of resveratrol on limbic hypothalamous-pituitary-adrenal gland axis (L-HPA)

The L-HPA axis is an important system increasing survival potential against psychological problems. Glucocorticoid hormones are the eventuall products of this axis acting on several systems such as the brain. Physical and psychological challenges can activate the HPA axis reflexively leading to production of corticotrophin releasing hormone (CRH), ACTH hormone, and thereby glucocorticoid secretion [103]. Although their beneficial effects, the prolonged releasing of glucocorticoids may result in multiple metabolic and psychological problems [104]. Evidence showed that hyperactivity of glucocorticoid negative feedback because of L_HPA dysregulation may be associated with some neuroendocrinological abnormalities [105]. On the other hand, the rapid elevation of brain-derivative neurotrophin factor (BDNF) expression precedes the activation of CRH in response to stress [106]. Moreover, BDNF is potentially involved in the regulation of HPA axis activity [107]. The L-HPA axis is reported as on of the main targets of resveratrol in anxiety disorder. For instance, the influence of trans-resveratrol on fear memory impairment and anxiety-like behaviors evaluated in a time-dependent sensitization (TDS) procedure-induced post-traumatic stress disorder (PTSD) model of mice. Trans-resveratrol alleviated abnormalities of L-HPA axis as well as the expressions of adrenal gland, corticotrophin-releasing factor levels, and glucocorticoid receptor in the hypothalamus, hippocampus, and amygdala. Moreover, trans-resveratrol elevated phosphorylation of cAMP response element binding protein (pCREB) and BDNF concentrations [108]. Similarly, another work detected the effect of trans-resveratrol on anxiety-like behavior and neuropathic pain in PTSD in vivo model induced by single-prolonged stress. Trans-resveratrol treatment led to decreased cold and mechanical allodynia, abnormalities of limbic hypothalamus–pituitary–adrenal axis. Furthermore, trans-resveratrol elevated protein kinase A, phosphorylated CREB protein, and BDNF [109].

#### The effect of resveratrol on inflammation and sirtuin regulation

Several studies claimed that inflammation can induce the progression of anxiety and depression [110, 111]. In addition the increased levels of inflammatory cytokines in the brain has been reported in mice models of anxiety [112]. One of the main factors promoting inflammation and proinflammatory cytokines expression is nuclear factor kappa B (NF-kB). Multiple studies indicated that resveratrol treatment could alleviate anxiety- and depression-like behaviors by inhibiting the NF-kB activation in the hippocampus [89].

In recent decades the epigenetic mechanisms have been suggested to be involved in the etiology of mood disorders such as anxiety, depression and related diseases [113–115]. Various epigenetic regulators induc epigenetic mechanisms leading to alteration in gene function. Sirtuins are the examples of these regulators known as class III HDACs [116]. Resveratrol is known as a potential sirtuin activator shown to be effective in recovery of hyper-anxiety in [117]. there are some evidence explore the effect of resveratrol on inflammation as well as sirtuin levels in anxiety. For instance, a recent study examined whether resveratrol ameliorates anxiety induced by ovariectomy in mice via suppression of inflammation. The authors reported that anxiety-like behaviors were significantly diminished by resveratrol administration. Additionally, resveratrol was able to decrease the activation of microglia in the hippocampal dentate gyrus as well as hippocampal levels of NLRP3, and NF-kB increased by estrogen deficiency. Furthermore, resveratrol inhibited cytokine production by boosting Sirt1 concentrations counteracting estrogen deficiency and therefore, its psycho-behavioral consequences [85]. Epidemiological evidence revealed that depression, anxiety and associated mental disorders may incidence as diabetes comorbidities. In another study pre-diabetic rats with hyper-anxiety behavior were used to investigate the mechanisms of these comorbidities. Rats were administered with resveratrol and metformin along with fructose. Resveratrol represented more effectiveness both in metabolic and anxiety disorders than metformin. Moreover, resveratrol was showed to upregulate some nuclear sirtuins including Sirt1 and Sirt7 decreased in the striatum of pre-diabetic rats. Thus, it was concluded that resveratrol might alleviate pre-diabetes-related hyper-anxiety via regulation of sirtuins [118].

Magaji et al., also found anxiolysis and anti-psychotic effects of 14-days treatment of resveratrol in mice model of anxiety and schizophrenia [119]. Pterostilbene is a natural analog of resveratrol with similar effects, however its neurological functions have not been fully explored. An investigation demonstrated that pterostilbene could represented anxiolytic function at 1 and 2 mg/kg doses, however this effect was not observed at higher doses. Thus, this was concluded that pterostilbene might be beneficial for the treatment of anxiety disorder as the same as resveratrol [120].

Table 2 indicates summarized data of studies evaluated role of resveratrol for treatment of anxiety.

**Table 2:** Animal studies that investigated the therapeutic effects of resveratrol on anxiety

Disease	Dose of resveratrol	Model	Findings	Publish year	Refs.
PTSD-induced anxiety	10, 20 and 40 mg/kg	In vivo	Alleviate L-HPA axis, corticotrophin-releasing factor levels, and glucocorticoid receptor in the hypothalamus, hippocampus, and amygdala, increased pCREB and BDNF	2018	[108]
PTSD-induced anxiety	40 mg/kg	In vivo	Alleviate L-HPA axis, elevated protein kinase A, phosphorylated CREB protein, and BDNF	2020	[109]
Anxiety	200 and 400 mg/kg	In vivo	Anxiolysis and anti-psychotic effects	2017	[119]
Ovariectomy-induced anxiety	20 mg/kg	In vivo	Decreased anxiety-like behaviors, decreased activation of microglia in the hippocampal dentate gyrus, hippocampal levels of NLRP3, and NF-kB, inhibited cytokine production, increased Sirt1 concentrations	2019	[85]
Diabetes-induced anxiety	10 mg/kg/day	In vivo	Increased Sirt1 and Sirt7	2016	[118]
Anxiety	1 and 2 mg/kg	In vivo	Anxiolytic function	2013	[120]

**Fig. 2:**
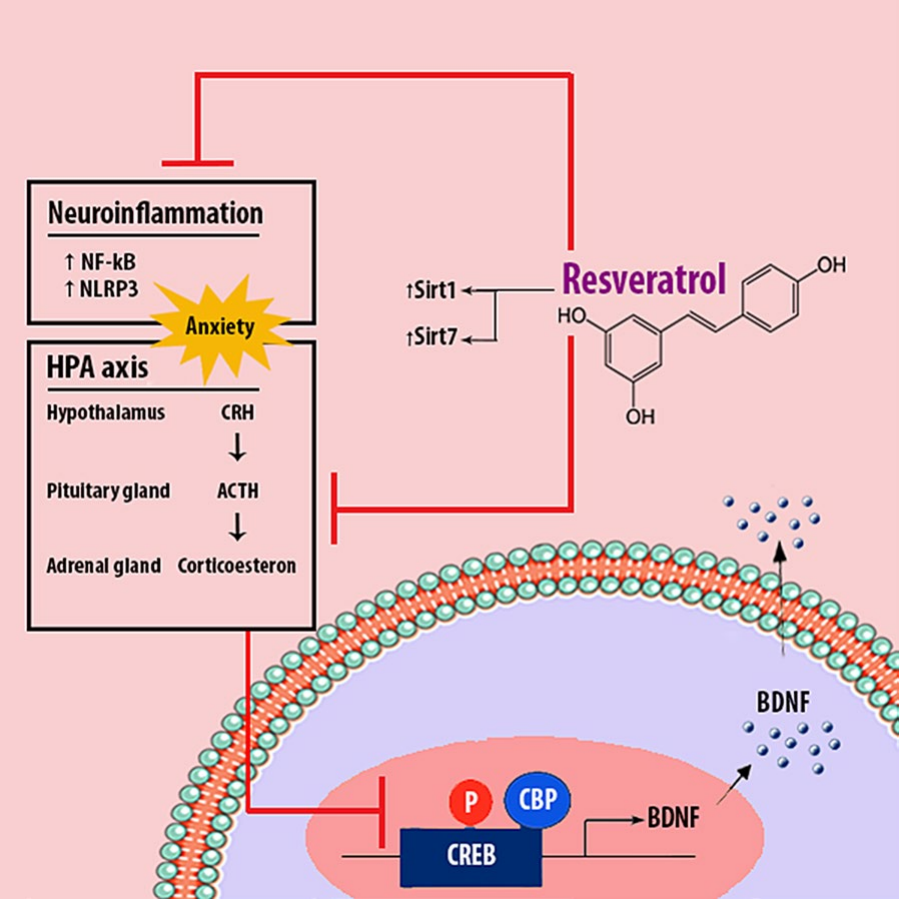
A schematic representation of resveratrol function in the treatment of Anxiety

The effects of resveratrole on pathways complicated in anxiety were indicated in Fig. 2.

### ASD

ASD is related to neural and synaptic deficits. The pathophysiology of ASD has been shown to be associated with oxidative stress, immune dysfunction, and excitation-inhibition imbalance. ASD is characterized by impairments in repetitive and social interactions, communication, restricted and stereotyped-like behavior. Multiple study have been conducted to investigate the effect of resveratrol and its mechanisms of action on ASD. In an in vivo study rodent model of autism induced by prenatal exposure to valproic acid was used for evaluating the effects of resveratrol on social behaviors. This study showed that resveratrol alleviated impairment of social behavior [121]. In contrast, a clinical trial showed no significant influence of resveratrol on patients with ASD. Resveratrol at dose of 250 mg twice per day was administered by the patients for 10 weeks. Irritability, lethargy/social withdrawal, stereotypic behavior, and inappropriate speech in resveratrol group were the same as the placebo group. Hyperactivity/non-compliance score was statistically decreased in the resveratrol group compared to the placebo group. Overall, this study found no considerable effect for resveratrol treatment on irritability of ASD patient while it could alleviate hyperactivity/non-compliance of ASD cases [122].

#### The effect of resveratrol on oxidative stress and inflammation

Chemokine receptors have been indicated to play important role in CNS involved in the progression of many neuro-inflammatory disorders. Interestingly, matrix metalloproteinases, pro-inflammatory cytokines, oxidative stress and mitochondrial dysfunction are the potential factors triggering neuro-inflammation resulting in neural dysfunction all of which are involved in ASD pathogenesis. Evidence supports the anti-inflammatory and andtioxidant effects of resveratrol in ASD. For example, the effect of resveratrol was assessed on neuroinflammatory model of rats with ASD. Resveratrol treatment led to a significant amelioration of behavioral, biochemical, molecular, sensory, and neurological deficits via inhibiting oxidative stress, mitochondrial dysfunction, MMP-9 and TNF-α expression in a dose dependent manner [10]. Another work demonstrated that resveratrol upregulated the gene expressions of Sirt1 and early growth response factor 3 (Egr3) in mice with autism leading to amelioration of their social behavior [123]. Xie et al., [124] hypothesized that exposure to prenatal progestin may enhance ASD development ameliorating by resveratrol treatment. Prenatal exposure of progestin in pregnant mice reduced estrogen receptor β (ERβ) expression in the amygdala leading to autism-like behavior. Prenatal and postnatal treatment with resveratrol activated ERβ via histone and DNA demethylation on ERβ promoter as well as reduced oxidative stress, mitochondrial dysfunction, and lipid metabolism in the brain therefor, alleviated autism-like behavior.

#### The effect of resveratrol on neuroimmune regulation

Immune dysregulation has been found to take role in the development of ASD and multiple markers of system neuroinflammation have been reported in ASD [63].

The role of immune abnormalities in the develop-ment of autism is beginning to emerge. Immune abnormalities also found in children with ASD at a young age [125]. In addition, the gene expression and function of immune cells were recognized to be dysregulated in ASD [126]. Moreover, several chemokines and cytokines increased in children with ASD [127]. Dysregulation of T helper (Th1, Th2, and Th17) and T regulatory cells transcription factor signaling were also found in autistic children (Ahmad et al. 2017b). Toll-like receptors (TLRs) are widely expressed in immune cells in the brain playing an essential role in neurophysiology [127]. Moreover, evidence showed the distribution of LTRs expression in the brain during neurocysticercosis [128]. Resveratrol is able to improve neuroimmune dysregulation by suppressing the inflammation-related signaling pathways. The BTBR T^+^ Itpr3^tf^/J (BTBR) autistic mouse model showing impaired juvenile play, social behavior, and low reciprocal social interaction was used for assessing resveratrol effects on autism treatment. The results indicated that CD4^+^TLR2, CD4^+^TLR3, CD4^+^TLR4, CD4^+^NF-κB^+^, and CD4^+^iNOS^+^ levels were decreased in spleen cells followed by resveratrol treatment. In addition, the gene expressions and protein levels of TLR2, TLR3, TLR4, NF-κB, iNOS, and COX-2 were reduced in the brain tissue after resveratrol intervention [129]. Another study detected the role of resveratrol in improving of the dysregulation of transcription factor in two models of autistic mice including BTBR mice and C57BL/6 (B6) mice. In both types, resveratrol led to upregulation of Foxp3^+^ and reduction of T-bet^+^, GATA-3^+^, and IL-17A^+^ expression and protein levels in CD4^+^ cells, spleen, and brain tissues in comparison to control groups [130]. Prenatal exposure to valproic acid may lead to ASD incidence. Pregnant rats were treated with resveratrol along with valproic acid. The results showed that resveratrol could prevent all mechanisms initiated by valproic acid suggesting to be a potential protective agent for ASD incidence [131]. Evidence indicated that JAK-STAT signaling pathway is related to some neurological diseases. A recent work showed that resveratrol was able to decrease IL-6^+^, TNF-α^+^, IFN-γ^+^, and STAT3^+^ in CD4^+^ spleen cells of autistic mice compared to control group. The result of this work also revealed that resveratrol administration reduced the genes expression and protein levels of TNF-α, IL-6, IFN-γ, JAK1, and STAT3 in the brain tissue [132]. Previous research declaimed that exposure to maternal hormone may develop ASD. An investigation evaluated the influence of resveratrol on the C–C chemokine receptor (CCR) and C-X-C motif chemokine receptor (CXCR) (CCR3^+^, CCR5^+^, CCR7^+^ and CCR9^+^, CXCR3^+^ and CXCR5^+^) in cluster of differentiation 4-positive (CD4^+^) T cells in the spleen and brain tissues of BTBR mice. Resveratrol treatment resulted in reduction of CCR and CXCR expression in CD4^+^ T cells in spleen and brain tissues in comparison with control group. Thus, resveratrol could decreased the chemokine receptors providing a unique goal for therapeutic strategies for autism [133].

This data of investigations assessing resveratrol effects for curing of ASD disorders summarized in Table 3.

**Table 3:** Studies that investigated the therapeutic effects of resveratrol on ASD

Disease	Dose of resveratrol	Model	Findings	Publish year	Ref
Animal stusies					
Valproic acid-induced ASD	3.6 mg/kg	In vivo	Improved social behavior	2014	[121]
Oxytocin receptor gene knockout (*Oxtr*-KO) and valproic acid-induced ASD	30 mg/kg	In vivo	Upregulated Sirt1 and Egr3, improved social behaviors	2020	[123]
BTBR-induced ASD	20 and 40 mg/kg	In vivo	Decreased levels CD4^+^TLR2^+^, CD4^+^TLR3^+^, CD4^+^TLR4^+^ CD4^+^NF-κB^+^, and CD4^+^iNOS^+^ in spleen cells, decreased TLR2, TLR3, TLR4, NF-κB, iNOS, and COX-2 in the brain tissue	2018	[129]
Valproic acid-induced ASD	3.6 mg/kg	In vivo	Prevented ASD-like behaviors	2018	[131]
B6- and BTBR-induced ASD	20 and 40 mg/kg	In vivo	upregulated Foxp3^+^, reduced T-bet^+^, GATA-3^+^, and IL-17A^+^ expressions and protein levels in CD4^+^ cells, spleen, and brain tissues	2017	[130]
BTBR-induced ASD	20 and 40 mg/kg	In vivo	Decreased levels of IL-6^+^, TNF-α^+^, IFN-γ^+^, and STAT3^+^ in CD4^+^ spleen cells and brain tissue	2017	[132]
Decreased ERβ- induced ASD	20 mg/kg	In vivo	activated ERβ, decreased oxidative stress, mitochondrial dysfunction, and lipid metabolism in the brain	2018	[124]
BTBR-induced ASD	20 and 40 mg/kg	In vivo	Decreased CCR and CXCR expression in CD4^+^ T cells in spleen and brain tissues	2016	[133]
ASD	5, 10 and 15 mg/kg	In vivo	Inhibited oxidative stress, mitochondrial dysfunction, MMP-9 and TNF-α expression	2017	[10]
Human studies					
ASD	250 mg twice per day	Human	Decreased hyperactivity/non-compliance score	2020	[122]

**Fig. 3:**
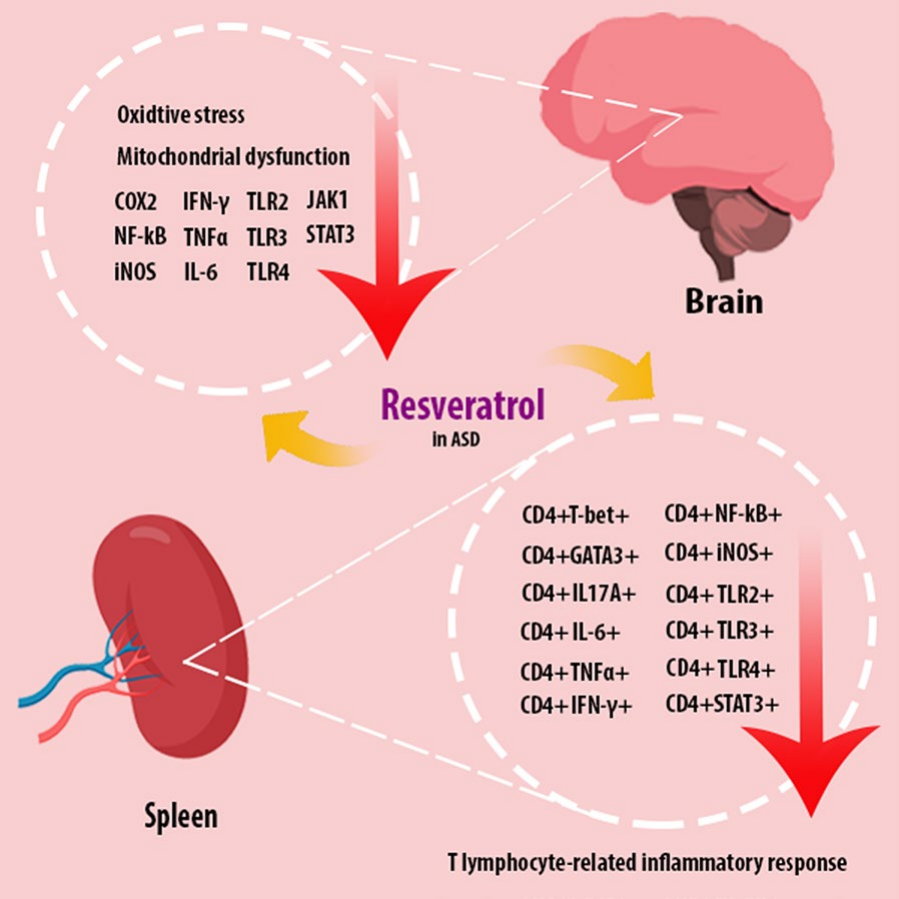
A schematic representation of resveratrol function in the treatment of ASD

The mechanisms of function of resveratrol in ASD were shown in Fig. 3.

### Schizophrenia

Schizophrenia is characterized by psychotic symptoms and cognitive impairments. Some studies revealed that resveratrol might be potential in treatment of schizophrenia due to its neuroprotective activities. Recently, an in vivo study was conducted to evaluate antipsychotic properties of resveratrol in schizophrenia. Schizophrenic mice were pretreated with resveratrol followed by behavioral tests on the 15th day. Anti-psychiotic and anxiolytic functions of resveratrol were determined by hole board and staircase tests as well as stereotypy-induced apormorphine and swim-induced groomin testes, respectively. The results indicated that resveratrol administration led to a significant reduction in the mean episodes of rearing as well as the mean climbing scores while it did not considerably decrease the mean number of head dips. Thus, it was concluded that resveratrol could represent anxiolytic and anti-psychotic influence in mice model [119]. Recent data reported that schizophrenia are commonly overweight or obese with many metabolic comorbidities. Moreover, these patients have a high mortality rate and life expectancy due to cardiovascular problems. In an in vivo research, resveratrol was administrated to neonatal rats with schizophrenia-like behavior in order to explore its effect on the alleviation of cognitive and motor deficits as well as associated molecular alterations. Findings showed that resveratrol contributed to the expressions of SIRT1 and BDNF as well as amelioration of oxidative stress [134].

Common antipsychotic drugs used for schizophrenia treatment may cause motor disorders such as tardive dyskinesia. In a recent study the influence of resveratrol administration on behavioral alterations result from chronic treatment with fluphenazine in rats was determined. The treatment of fluphenazine led to decreased body weight gain and the number of rearing and crossing while co-administration with resveratrol led to none of these changes. Fluphenzine also elevated the frequency and intensity of vacuous chewing movments (VCMs) whereas resveratrol cotreatment decreased the VCMs. Additionally, the number of VCMs was negatively correlated with monoamine oxidase-B (MAO-B) in the stratum of rats [135]. It has been reported that the infection of several agents such as bacteria, arboviruses, or some protozoans during pregnancy may be related to increased risk of schizophrenia. Immunological mechanisms activated by infection may impair neural progenitor and CNS functions. In a study, pregnant mice induced by maternal immune activation (MIA) were treated with resveratrol. Behavioral tests were performed on the day 45 after birth. Results showed that resveratrol administration ameliorated the hyperlocomotion as well as social behavior. Resveratrol inhibited the influence of MIA on the expressions of Syn1 and Olig1 in the cortex and hippocampus therefore impacting on synaptic and oligodendrogenesis mechanisms [136]. In a randomized, double-blind controlled trial, patients with schizophrenia enrolled to assess the effect of resveratrol on metabolic parameters including serum glucose and cardiovascular risk factors. In summary, resveratrol intake led to no significant changes. However, lipid profile exacerbated in placebo group. It was concluded that resveratrol administration might prevent lipid profile worsening [137]. In another randomized, double blind, and controlled trial patients suffering from schizophrenia were subjected to receive resveratrol supplementation or placebo for 1-month. Neuropsychology performance and psychology severity were evaluated. The results indicated that neuropsychology performance criteria including episodic and work memory, capacity of attention and concentration, interference measures, inhibitory control, mental flexibility, and selective attention as well as psychology severity did not improved after 1 month of resveratrol treatment [138]. Table 4 shows a summary of these studies.

**Table 4:** Studies that investigated the therapeutic effects of resveratrol on schizophrenia

Disease	Dose of resveratrol	Model	Findings	Publish year	Ref
Animal studies					
Schizophrenia	200 and 400 mg/kg	In vivo	reduced mean episodes of rearing and mean climbing scores	2017	[119]
MK-801-induced schizophrenia	40 mg/kg or 80 mg/kg	In vivo	Increased expression of SIRT1 and BDNF, decreased oxidative stress	2020	[134]
Schizophrenia	20 mg/kg	In vivo	Decreased VCMs	2017	[135]
MIA-induced schizophrenia	40 mg/kg	In vivo	Ameliorated hyperlocomotion and social behavior	2020	[136]
Human studies					
Schizophrenia	200 mg/day	Human	No significant changes	2016	[137]
Schizophrenia	200 mg/day	Human	No significant changes	2016	[138]

## Conclusions

In current article, we reviewed recent achievements of chitosan-based drug delivery systems for curing of the mental disorders including depression, anxiety, ASD, and schizophrenia. Mental disorders are multifactorial diseases requiring drugs that are able to effective on many brain targets. Resveratrol have been shown to have pleiotropic neuroprotective effects appear to be a potential candidate for using in treatment of mental disorders. Although, its clinical effectiveness and application are not clear yet. Resveratrol is able to interfere with multiple mechanisms complicated in the beginning stages of pathology of mental disorders preventing or slowing down them. However, the establishment of the effective dose, standardized preparations, and improvement of bioavailability are the primary points which should be considered. In recent decades, resveratrol has received great attentions for curing mental disorders due to its various therapeutic features such as antioxidant and anti-inflammatory effects and many animal studies have been conducted in this way, however clinical investigations are limited. Thus, the potential features of resveratrol in mental disorders treatment should be examined in long-term interventions in clinical manner.
